# Case Report: Intentional massive melatonin ingestion (450 mg) in an adolescent female

**DOI:** 10.3389/ftox.2026.1958318

**Published:** 2026-10-16

**Authors:** Sepehr Miran, Bahar Amiri, Peyman Astaraki, Arian Karimi Rouzbahani

**Affiliations:** 1 Department of Surgery, Monash University, Melbourne, VIC, Australia; 2 Western Clinical Trials, Western Urology, Melbourne, VIC, Australia; 3 Surgery Education and Researching Network (SERGN), Universal Scientific Education and Research Network (USERN), Melbourne, VIC, Australia; 4 Razi Herbal Medicines Research Center, Lorestan University of Medical Sciences, Khorramabad, Iran; 5 Department of Internal Medicine, Shahid Rahimi Hospital, Lorestan University of Medical Sciences, Khorramabad, Iran; 6 Surgery Education and Researching Network (SERGN), Universal Scientific Education and Research Network (USERN), Khorramabad, Iran

**Keywords:** adolescent, case report, CYP1A2, intoxication, literature review, melatonin, poison severity score, suicide attempt

## Abstract

Over-the-counter (OTC) melatonin is widely perceived as benign, yet its unregulated, high-dose formulations create an accessible means of self-harm for adolescents, and reports of intentional overdose have risen alongside a well-documented surge in pediatric exposures. We report a 16-year-old female who intentionally ingested 450 mg of melatonin (90 × 5 mg tablets), a 90-fold supratherapeutic dose, and synthesize this case against the published literature on melatonin overdose, suicidality, and toxicokinetics. She presented 2.5 h post-ingestion with marked somnolence (Glasgow Coma Scale 13/15) and heart rate of 62 beats/min. Venous blood gas showed a mild, respiratory-compensated metabolic acidosis (pH 7.34, HCO3 15.6 mmol/L), with mild hyponatremia and stress leukocytosis. Hepatic, renal, and coagulation studies and a 12-lead electrocardiogram were normal, and a comprehensive toxicology screen was negative, consistent with an isolated exposure (Poison Severity Score Grade 1, Minor). She was medically cleared within 14.5 h of ingestion but, given persistent suicidal ideation on psychiatric evaluation, was transferred to inpatient psychiatric care at 20.5 h post-ingestion. This case, together with a review of published case reports, cohort data, and toxicokinetic evidence, indicates that massive isolated melatonin ingestion typically produces self-limited sedation, consistent with saturable hepatic CYP1A2-mediated clearance, alongside mild, transient metabolic derangement without organ injury. Population-level and case-level evidence converge, however, on a critical message for clinicians: low physiological toxicity does not indicate low psychiatric risk, and every adolescent presenting after intentional OTC chronobiotic ingestion warrants formal psychiatric evaluation regardless of how benign the medical course appears.

## Introduction

Exogenous melatonin (5-methoxy-N-acetyltryptamine) is a widely available, over-the-counter (OTC) chronobiotic marketed for circadian sleep-wake disorders. Melatonin is regulated as a dietary supplement in most countries and it is largely exempt from the premarket safety oversight applied to licensed hypnotics. Household access to high-dose, bulk formulations is essentially unrestricted. This regulatory status, combined with aggressive retail marketing, has driven a dramatic rise in pediatric and adolescent melatonin exposure. A surveillance of the United States National Poison Data System showed a 530% increase in pediatric melatonin ingestions between 2012 and 2021, 84.4% of the 260,435 exposures identified remaining asymptomatic, five cases requiring mechanical ventilation, and two deaths (Lelak et al., 2022). In 2020, melatonin became the single substance most frequently reported to United States poison control centers (Lelak et al., 2022).

Against this epidemiological backdrop, melatonin is increasingly implicated not only in unintentional pediatric exposure but in intentional self-poisoning among adolescents and adults, particularly those with underlying mood or sleep disturbance. Case reports and small series published over the past 3 decades describe intentional melatonin overdoses ranging from tens to nearly a thousand milligrams, with marked sedation as the dominant clinical feature and supportive care as the mainstay of management (Chung, 2001; Holliman and Chyka, 1997; Gutierrez Higueras et al., 2022; Tripathi et al., 2024). A recurring diagnostic and interpretive challenge in this literature is distinguishing the toxicity of melatonin itself from that of co-ingestants. The only published suicide death involving melatonin resulted from combined diphenhydramine–melatonin toxicity rather than melatonin ingestion alone (Zimmerman et al., 2023). Furthermore, a case of transient Brugada-pattern electrocardiographic change was reported only in the context of a concomitant alcohol binge (Hassan et al., 2023).

What is unique about this case is the combination of a very high, quantified dose (450 mg), a negative comprehensive co-ingestant screen, and acid-base and hematologic documentation. This data remain sparse for isolated melatonin ingestions exceeding 400 mg. Our report has three main objectives: (I) to detail the clinical, acid-base, and hematologic manifestations of this ingestion; (II) to situate the case within the broader literature on melatonin self-poisoning, population-level suicidality signals, toxicokinetics, and product-content variability; and (III) to illustrate, through this patient’s progression, that a substance’s low physiological lethality does not equate to low psychiatric risk.

## Case description

A 16-year-old female (weight 54 kg [119 lb]; height 160 cm; BMI 21.1 kg/m^2^) presented to the emergency department with an altered level of consciousness approximately 2.5 h after an intentional ingestion of 90 melatonin tablets. Psychosocial assessment confirmed the event was a suicide attempt. She had ingested three 30-tablet packages of 5 mg melatonin (a commercial over-the-counter product), a cumulative dose of 450 mg at home; this figure reflects labeled tablet content rather than a directly measured ingested dose, given well-documented variability in commercial melatonin product labeling (see Discussion). Her past medical history was unremarkable, with no prior deliberate self-harm, suicide attempts, psychiatric hospitalization, or toxicological exposure. She had a background of complex family dynamics and socioeconomic adversity from an early age and a recent deterioration in financial circumstances was believed to represent an additional psychosocial stressor. Further detailed psychological and psychosocial information is not reported because of the confidential nature of the patient’s ongoing psychiatric care. She was not prescribed any regular medication, including psychotropic agents such as selective serotonin reuptake inhibitors (SSRIs). The provisional diagnosis at presentation was melatonin poisoning.

On presentation she was markedly somnolent and lethargic, drifting to sleep when undisturbed but easily arousable to verbal stimuli, consistent with central nervous system depression from massive melatonin MT1/MT2 receptor agonism. Glasgow Coma Scale was assessed to be 13/15 (eye opening 3, to voice; verbal response 4, confused; motor response 6, obeys commands). Vital signs were: blood pressure 110/70 mmHg, heart rate 62 beats/min, respiratory rate 12 breaths/min, oxygen saturation 95% on air, temperature 36.4 °C. The airway was intact, heart sounds were normal, lungs were clear, and the abdomen was soft non-tender with normal bowel sounds.

### Diagnostic assessment, therapeutic intervention, follow-up, and outcomes

A comprehensive workup excluded co-ingestion and end-organ injury (Table 1). Venous blood gas showed a mild, respiratory-compensated metabolic acidosis (pH 7.34, pCO2 29.0 mmHg, pO2 56 mmHg, HCO3 15.6 mmol/L, base excess −8.8 mmol/L) with a normal anion gap (13.1 mEq/L). Serum sodium was mildly low (134.3 mmol/L) and chloride was 105.6 mmol/L; potassium, urea, creatinine, and blood glucose were normal. Hepatic transaminases were normal. While alkaline phosphatase (305 IU/L) was within the local laboratory’s reference range, age-specific pediatric intervals suggest this may represent a mild elevation of uncertain clinical significance. Coagulation studies were normal. A mild leukocytosis with neutrophilic predominance was consistent with a physiological stress response. Hemoglobin, hematocrit, and platelet count were normal. Urine and serum toxicology screening was negative for acetaminophen, salicylates, ethanol, benzodiazepines, opioids, tricyclic antidepressants, amphetamines, diphenhydramine/antihistamines, and cocaine, consistent with an isolated melatonin exposure based on the substances tested. A 12-lead electrocardiogram was unremarkable, with no conduction abnormality detected.

**TABLE 1:** Complete laboratory and diagnostic panel at admission.

Parameter	Admission value/ Finding	Normal reference range
Venous pH	7.34	7.31–7.41 (venous)
Venous pCO2	29.0 mmHg	41–51 mmHg (venous)
Venous pO2	56 mmHg	30–50 mmHg (venous)
Venous HCO3–	15.6 mmol/L	22–29 mmol/L
Base excess (BEb)	−8.8 mmol/L	−2 to +2 mmol/L
Venous lactate	2.2 mmol/L	0.5–2.0 mmol/L
Serum sodium	134.3 mmol/L	135–145 mmol/L
Serum chloride	105.6 mmol/L	98–107 mmol/L
Anion gap	13.1 mEq/L	8–16 mEq/L
Serum potassium	4.3 mmol/L	3.5–5.0 mmol/L
Blood urea	27 mg/dL	18–45 mg/dL
Serum creatinine	0.71 mg/dL	0.6–1.3 mg/dL
Blood glucose (random)	92 mg/dL	70–140 mg/dL (random, non-fasting)
AST (SGOT)	20 IU/L	<31 IU/L
ALT (SGPT)	9 IU/L	<31 IU/L
Alkaline phosphatase (ALP)	305 IU/L	64–305 IU/L (female)
White blood cell count	12.2 × 10^9^/L (H)	4.0–10.0 ×10^9^/L
Neutrophils/ Lymphocytes/ Monocytes	82%/ 14%/ 4%	40%–75%/ 20%–45%/ 2%–10%
Hemoglobin	13.2 g/dL	12–15 g/dL
Hematocrit	38.8%	35%–45%
Red blood cell count	4.43 million/µL	4.1–5.1 million/µL
MCV/ MCH/ MCHC	87.6 fL/ 29.8 pg/ 34.0 g/dL	80–100 fL/ 27–33 pg/ 31–36 g/dL
Platelet count	213 × 10^9^/L	150–450 ×10^9^/L
RDW-CV/ MPV	11.9%/ 9.3 fL	11.0%–14.5%/ 5.0–12.0 fL
PT/ INR/ PTT	12.2 s/ 0.96/ 33.0 s	11–15 s/ 0.7–1.2/ 20–40 s
Additional diagnostic findings	Result	–
Urine/serum toxicology screen	Negative (acetaminophen, salicylates, ethanol, benzodiazepines, opioids, tricyclic antidepressants, amphetamines, diphenhydramine/antihistamines, cocaine)	–
12-Lead ECG	Normal (no conduction abnormality)	–
Poison severity score (PSS)	Grade 1 (minor)	–

VBG, venous blood gas.

Given the mild, transient symptoms without end-organ toxicity, the ingestion was classified Poison Severity Score Grade 1 (Minor) (Persson et al., 1998). This classification reflects predominantly minor findings on the PSS nervous system axis (drowsiness with preserved arousability to voice, corresponding to the Minor grade) and metabolic axis (HCO3 15.6 mmol/L, within the Minor range of 15–20 mmol/L). Her heart rate of 62 beats/min falls at the lower boundary of the pediatric threshold used by the PSS for bradycardia (60–80 beats/min), but was an isolated, non-progressive, asymptomatic finding without hemodynamic compromise and was not clinically identified or managed as bradycardia, consistent with an overall Minor (Grade 1) classification. Medical management for this patient was mainly supportive and as follows: strict nil-by-mouth status and complete bed rest to reduce aspiration risk given her depressed neurologic status, 0.9% NaCl (1 L IV stat, then additional boluses per a three-times-daily [TDS] order) to correct hyponatremia and maintain intravascular volume, one vial of 50% dextrose (given for a mildly low bedside glucose reading obtained on arrival, which preceded the subsequently normal laboratory venous glucose of 92 mg/dL), intravenous pantoprazole and ondansetron (associated nausea and risk of aspiration), a Foley catheter for strict intake/output monitoring, and vital signs every 6 h. Supplemental oxygen via nasal cannula was ordered as a threshold intervention should SpO2 fall below 92%. However, this was ultimately not required.

By 14.5 h post-ingestion (12 h after presentation), somnolence had fully resolved (GCS 15), she was tolerating oral fluids, intravenous fluids were discontinued, and she was medically cleared by the toxicology/attending team. Repeat venous blood gas confirmed resolution of the acidosis (pH 7.38), and hyponatremia (sodium 138.0 mmol/L) and leukocytosis (WBC 8.4 × 10^9^/L) had also normalized. At 16.5 h post-ingestion, a comprehensive bedside evaluation by the consult-liaison psychiatry team revealed ongoing suicidal ideation. Further details of this evaluation, including specific psychiatric history and diagnosis, are not reported here in order to protect patient confidentiality. Given this finding together with the high lethality intent reflected by the 90-tablet ingestion, she was not discharged home; at 20.5 h post-ingestion, she was transferred directly from the medical ward to a secure inpatient adolescent psychiatric unit for acute stabilization, safety planning, and initiation of psychiatric treatment. Total medical (non-psychiatric) length of stay was 18 h (Table 2).

**TABLE 2:** Clinical timeline of episode of care.

Time post-ingestion	Clinical setting, findings, and interventions
0.0 h	Home: Intentional ingestion of 450 mg melatonin (three 30-tablet packages of 5 mg tablets) in a suicide attempt
2.5 h	Emergency department: presentation with altered consciousness, marked somnolence (GCS 13/15), and CNS depression. Provisional diagnosis: melatonin poisoning. Initial panel drawn (biochemistry, electrolytes, CBC, coagulation, VBG, drug screen, 12-lead ECG). NPO and complete bed rest instituted; IV normal saline, 50% dextrose, and pantoprazole administered; Foley catheter placed
2.5–14.5 h	ED/Medical ward: vitals monitored every 6 h SpO2 maintained above 92% on room air without supplemental oxygen. Course categorized as poison severity score grade 1 (minor)
14.5 h	Medical ward: somnolence fully resolved (GCS 15). Tolerating oral fluids; IV fluids discontinued. Serial VBG confirmed resolution of metabolic acidosis; hyponatremia and leukocytosis resolved. Medically cleared by the toxicology/attending team (12 h after ED arrival)
16.5 h	Bedside consult-liaison psychiatry evaluation (14 h after ED arrival); ongoing suicidal ideation identified
20.5 h	Transferred directly from the medical ward to a secure inpatient adolescent psychiatric unit for acute stabilization and safety planning. Total medical (non-psychiatric) length of stay: 18 h (from ED arrival)

## Discussion

### Strengths and limitations

This case adds detailed acid-base, and hematologic data, together with a precisely timed clinical course, to the sparse literature on massive, isolated melatonin ingestion. Its principal strength is that co-ingestion was considered unlikely following a negative comprehensive toxicology screen, allowing the observed acidemia, hyponatremia, and leukocytosis to be considered in the context of a likely isolated melatonin exposure rather than a mixed-substance ingestion. This is a distinction that, as reviewed below, is frequently unavailable in more severe or fatal published cases (Tripathi et al., 2024; Zimmerman et al., 2023). Its main limitation is that quantitative serum melatonin concentrations were not obtained, since specific assays are not routinely available in acute care settings; toxicokinetic inference therefore relies on dose, timing, and clinical course rather than measured levels, a limitation shared with much of the case literature discussed below. Chloride was measured and yielded a normal anion gap (13.1 mEq/L); lactate was mildly elevated (2.2 mmol/L; local reference range 0.5–2.0 mmol/L), consistent with a contribution from stress- or perfusion-related lactate production. However, this is not of a magnitude suggesting a clinically significant lactic acidosis or meaningfully widening the anion gap. Melatonin has no established direct biochemical mechanism for producing metabolic acidosis and saturation of CYP1A2 plausibly explains patient’s prolonged sedation but not the acid-base findings in this patient. The observed acidemia is most plausibly a nonspecific consequence of her profound sedation, reduced oral intake, possible mild hypoperfusion, and physiological stress in a lethargic, poorly responsive patient. Similarly, the hyponatremia and leukocytosis are best understood as nonspecific findings associated with this clinical picture rather than as mechanistically establish

ed, direct effects of melatonin.

### Comparison with the published case literature

Table 3 situates this case within the published literature on melatonin self-poisoning. The earliest cluster, reported by Chung (2001), described three patients who presented after suicide attempts by melatonin overdose (doses up to 60 mg), all with uneventful hospital stays. This finding was interpreted as early evidence of melatonin’s wide therapeutic index. Holliman and Chyka (1997) demonstrated that clinically apparent toxicity (lethargy, disorientation, CNS depression) can occur far below the doses seen in deliberate overdose, following a non-suicidal 24 mg preoperative dosing error. Gutierrez Higueras et al. (2022) described a 42-year-old woman who ingested 120 mg in a suicide attempt, developing drowsiness and mild hypothermia but discharged after 12 h of observation with subsequent psychiatric diagnoses of depressive mood disorder and chronic insomnia. Tripathi et al. (2024) reported a 16-year-old male who intentionally co-ingested 900 mg of melatonin with 5 mg of alprazolam, presenting with a Glasgow Coma Scale of 8/15 and regaining consciousness at approximately 32 h.

**TABLE 3:** Comparison of published melatonin overdose and self-harm case reports/series.

Author (Year)	Case type	Age/Sex	Dose & route	Co-ingestant(s)	Presentation	Outcome
Chung (2001)	Isolated overdose	3 adults	Up to 60 mg, oral (suicide attempts)	None (isolated)	Asymptomatic to mild drowsiness	Uneventful; discharged, psychiatric referral
Holliman and Chyka (1997)	Isolated overdose	66 years/o M	24 mg, oral (accidental dosing error)	None	Lethargy, disorientation, CNS depression	Uneventful recovery
Gutierrez Higueras et al. (2022)	Isolated overdose	42 years/o F	120 mg, oral (suicide attempt)	None (isolated)	Drowsiness, mild hypothermia (34 °C)	Discharged at 12 h; psychiatric diagnosis
Tripathi et al. (2024)	Co-ingestant overdose	16 years/o M	900 mg, oral (suicide attempt)	Alprazolam 5 mg	GCS 8/15, minimal responsiveness	Conscious at ∼32 h; psychiatric transfer
Zimmerman et al. (2023)	Co-ingestant overdose	Young adult (decedent)	Up to ∼480 mg (estimated)	Diphenhydramine	N/A (*postmortem*)	Death from combined DPH–melatonin toxicity
Hassan et al. (2023)	Co-ingestant overdose	37 years/o M	430 mg, oral (43 × 10 mg)	Alcohol (binge drinking)	Transient brugada type 2 ECG pattern	Resolved within a few hours; no intervention
Warren et al. (2016)	IV misuse	Adult	IV injection of crushed tablets (period of ∼2 years)	None specified	Recurrent infective endocarditis	Aortic valve replacement required
Badrfam and Zandifar (2026)	Isolated (therapeutic dose)	Adult	Single therapeutic dose	None	New-onset suicidal ideation	Resolved; causality confounded, not established
Labay et al. (2019)	*Postmortem* pediatric series	22 cases, mixed pediatric ages (forensic series)	Concentrations quantified via *postmortem* assessment	Variable	N/A (*postmortem*)	Melatonin detected; cause/manner often undetermined
Shimomura et al. (2019)	*Postmortem* pediatric (chronic)	Twin infant	∼40–50 mg/day oral (8–10 × 5 mg tablets daily, chronic)	None identified	Sudden unexplained death	*Postmortem* melatonin toxicology; interpretation challenging
Bishop-Freeman et al. (2022)	*Postmortem* pediatric series	7 infants/toddlers (2 months–3 years)	Variable	Variable	N/A (*postmortem*)	Melatonin detected; manner/cause frequently undetermined
Present case	Isolated overdose	16 years/o F	450 mg, oral (90 × 5 mg)	None (isolated; confirmed negative screen)	GCS 13/15, mild acidemia, leukocytosis	PSS grade 1; cleared 14.5 h post-ingestion; psychiatric transfer 20.5 h post-ingestion

Zimmerman et al. (2023) reported the only published suicide death in this literature: a young adult with markedly elevated *postmortem* melatonin concentrations who had co-ingested diphenhydramine, with death certified as due to combined toxicity, as a lethal threshold for melatonin alone remains undefined. Hassan et al. (2023) described a transient Brugada type 2 electrocardiographic pattern in a 37-year-old man after ingesting 430 mg of melatonin, during an alcohol binge, which resolved within a few hours without specific intervention. Our patient’s 12-lead ECG showed no such changes, suggesting the cardiac finding in that case may reflect the concomitant alcohol intoxication rather than ingestion of melatonin itself. Warren et al. (2016) described an unusual route of misuse, intravenous injection of crushed melatonin tablets, resulting in recurrent infective endocarditis requiring valve replacement, demonstrating that morbidity in that case arose from the route of administration rather than melatonin’s pharmacology. Badrfam and Zandifar (2026) described new-onset suicidal ideation shortly after a single therapeutic dose of melatonin in a patient with major depressive disorder, though active depression, insomnia, and psychosocial stressors were major confounders that left causal inference weak.

Our patient’s clinical course, marked but self-limited CNS depression, mild compensated acidemia, and full medical recovery within 14.5 h, sits between the mild, brief presentations described by Chung (2001) and Gutierrez Higueras et al. (2022) and the more severe, prolonged course of Tripathi et al.'s (2024) co-ingestion case. It also differs markedly from the fatal outcome reported by Zimmerman et al. (2023), which required a co-ingestant to reach lethality.

### Population-level suicidality signals

Beyond individual case reports, a Danish nationwide cohort study by Høier et al. (2022) followed 5,798,923 individuals, of whom 10,577 received melatonin. They found adjusted incidence rate ratios of 4.8 for suicide and 5.9 for suicide attempt among those treated with melatonin compared with those not treated. However, the authors emphasized that this association most plausibly reflects confounding by underlying psychiatric comorbidity rather than a direct pharmacologic effect. By contrast, a Swedish cohort study by Leone et al. (2023) examining melatonin initiation in youths with and without psychiatric disorders found that self-harm risk was highest in the month immediately preceding melatonin initiation and fell afterward, with an incidence rate ratio of 0.46 (95% CI 0.27–0.76) in the month following initiation among adolescent females with psychiatric disorders. The authors interpreted this pattern as most consistent with melatonin being prescribed at a moment of acute crisis that was already resolving, rather than melatonin itself altering self-harm risk in either direction. This further underscores that the population-level associations with melatonin use are heavily confounded by the underlying reasons for its prescription. Taken together with the single-dose ideation case of Badrfam and Zandifar (2026), this literature supports association rather than a causation relationship between melatonin treatment and suicidality, and underscores that melatonin use itself may be a marker of an already high-risk psychiatric population rather than a toxic driver of self-harm.

### Toxicokinetics and product-content variability

Oral melatonin normally has low, variable bioavailability (9%–33%) owing to extensive first-pass hepatic metabolism, reaching peak plasma concentration within 30–60 min at physiological doses (1–5 mg) and undergoing rapid elimination (half-life 1–2 h) via hepatic cytochrome P450 1A2 (CYP1A2), which accounts for over 90% of its clearance (Harpsøe et al., 2015; Zawilska et al., 2009). At a 450 mg dose, roughly 90 times a typical physiological dose, hepatic CYP1A2 capacity may plausibly become saturated, a hypothesized shift from first-order toward zero-order elimination kinetics that could prolong both systemic exposure and CNS depression observed in this patient (Figure 1). No serum melatonin concentrations were measured in this case, so this mechanism remains a proposed explanation rather than a directly demonstrated one; it is offered as one hypothesis consistent with the extended, roughly 12-h clinical recovery observed here despite melatonin’s ordinarily short half-life. Compounding this, independent laboratory analyses of commercial melatonin products have documented substantial label-content discrepancies, ranging from −83% to +478% of the stated dose, and identified serotonin contamination in a meaningful proportion of products. This can introduce further uncertainty into the true ingested dose in any reported overdose (Erland and Saxena, 2017). A large systematic review and meta-analysis of high-dose melatonin trials (≥10 mg) in adults found no detectable increase in serious adverse events in the low-risk-of-bias subset (rate ratio 0.88, 95% CI 0.52–1.50) though adverse events such as drowsiness, headache, and dizziness were modestly increased (rate ratio 1.40, 95% CI 1.15–1.69) (Menczel Schrire et al., 2022). A related systematic review similarly characterized melatonin’s adverse effect profile as generally mild (Besag et al., 2019).

**FIGURE 1:**
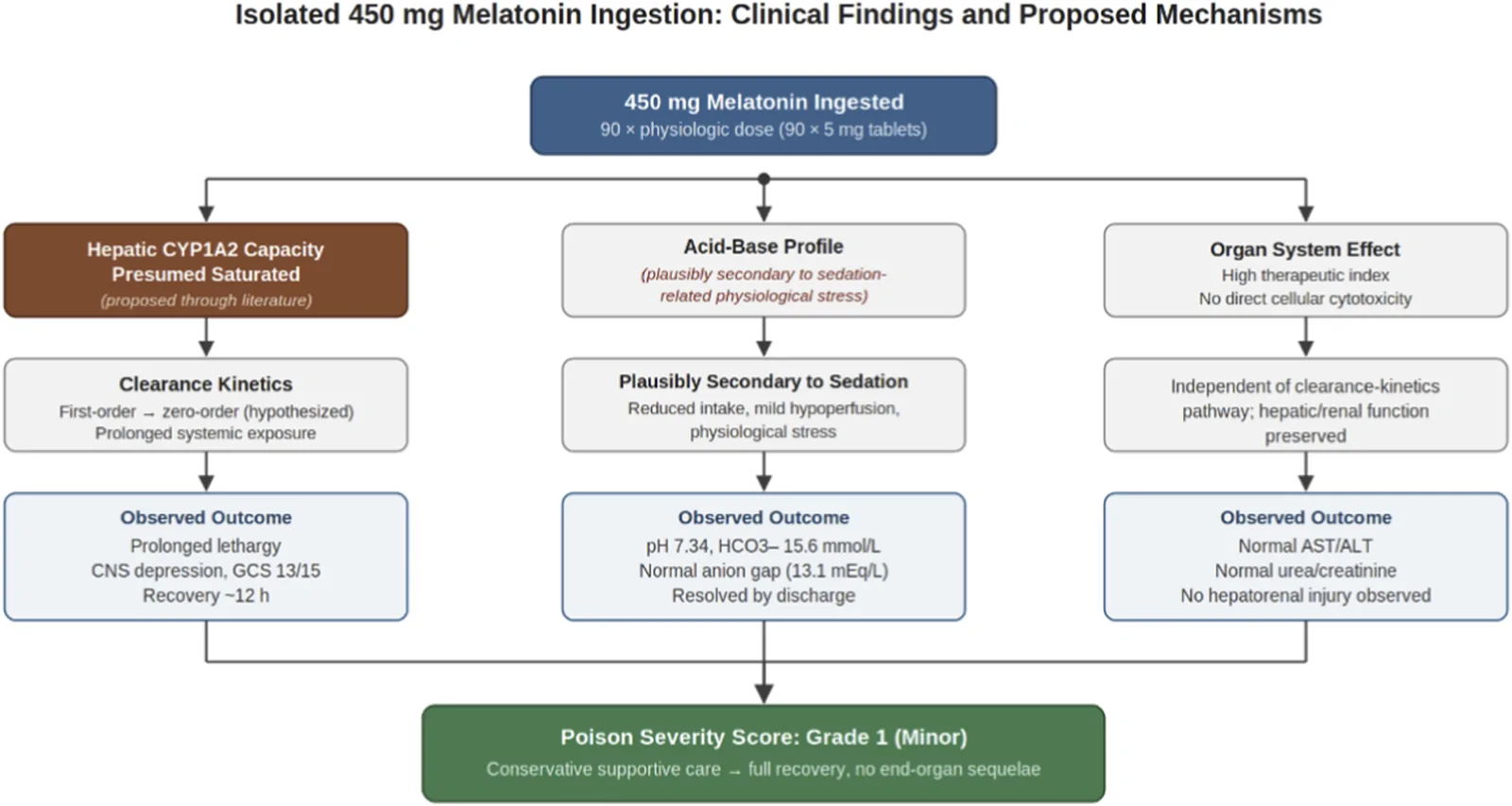
Clinical findings following isolated 450 mg melatonin ingestion are depicted here, organized by proposed mechanism. CYP1A2 saturation is a proposed explanation for prolonged sedation and clearance. Acid-base or hematologic findings observed in this patient are most likely a nonspecific consequence of sedation- or hypoperfusion-related stress.

### Pediatric safety signals and forensic considerations

Pediatric toxicovigilance and forensic literature reinforce that, while most exposures are benign, melatonin can be clinically and forensically significant in vulnerable populations. A recent clinical review characterized pediatric melatonin toxicity as predominantly mild, with gastrointestinal upset and drowsiness as the most common reported symptoms, while noting that product-variability concerns complicate the process of risk assessment in children (Shenoy et al., 2024). Forensic case series have documented markedly elevated *postmortem* melatonin concentrations in infant deaths (Labay et al., 2019; Shimomura et al., 2019; Bishop-Freeman et al., 2022). However, causal attribution in these cases is frequently classified as undetermined rather than proven melatonin poisoning, further reflecting the interpretive challenges of *postmortem* melatonin toxicology in infants. A national survey of melatonin use in young children (Kracht et al., 2026) and a systematic GRADE-based review of pediatric melatonin trials (Händel et al., 2023) both similarly conclude that serious adverse effects are rare in the therapeutic-use setting, in contrast to the acute overdose setting reviewed above.

### Clinical take-away

Consistent with the broader literature reviewed above, melatonin toxicity in isolation is expected to produce mild, self-limited physical symptoms while the central challenge illustrated by this case is psychiatric, not physical risk. In this case, deliberate consumption of three 30-tablet packages indicated clear intent and planning, and bedside evaluation subsequently confirmed ongoing suicidal ideation despite full medical recovery. The patient was accordingly transferred to inpatient psychiatric care rather than discharged home. Low physical toxicity can create false reassurance and clinicians should not equate the mild physiological severity of a melatonin ingestion with a low risk of future, potentially fatal, self-harm behavior. Every adolescent presenting after an intentional OTC chronobiotic ingestion should receive formal psychiatric evaluation before discharge, regardless of how benign the medical course appears.

## Data Availability

The original contributions presented in the study are included in the article/Supplementary Material, further inquiries can be directed to the corresponding author.
