# Supplementary material for: Case Report: Intentional massive melatonin ingestion (450 mg) in an adolescent female
Source: Front Toxicol. 2026 Oct 16;8:1958318. doi: 10.3389/ftox.2026.1958318 (PMC13719137; doi:10.3389/ftox.2026.1958318)
Supplement: Supplementary file 1 [file DataSheet1.pdf]

CARE Checklist of Information to Include When Writing a Case Report

Manuscript: Case Report: Intentional Massive Melatonin Ingestion (450 mg) in an Adolescent Female

Topic	Item	Checklist Item Description	Reported in Manuscript (Section)
**Title**	1	The diagnosis or intervention of primary focus followed by the words "case report"	Title page: "Case Report: Intentional Massive Melatonin Ingestion (450 mg) in an Adolescent Female"
**Key Words**	2	2 to 5 key words that identify diagnoses or interventions in this case report, including "case report"	Title page, Keywords line (includes "case report")
**Abstract (no references)**	3a	Introduction: What is unique about this case and what does it add to the scientific literature?	Abstract, opening sentences
	3b	Main symptoms and/or important clinical findings	Abstract, middle (GCS 13/15, VBG/acid-base findings, hyponatremia, leukocytosis)
	3c	The main diagnoses, therapeutic interventions, and outcomes	Abstract (Poison Severity Score Grade 1, Minor; supportive care; medically cleared at 14.5 h after ingestion; psychiatric transfer at 20.5 h)
	3d	Conclusion — What is the main "take-away" lesson(s) from this case?	Abstract, closing sentence
**Introduction**	4	One or two paragraphs summarizing why this case is unique (may include references)	Introduction section, paragraph beginning "What is unique about the present case..."
**Patient Information**	5a	De-identified patient specific information	Case Description, opening paragraph (age, sex, weight, height, BMI)
	5b	Primary concerns and symptoms of the patient	Case Description, opening paragraph (altered consciousness following intentional ingestion)
	5c	Medical, family, and psycho-social history including relevant genetic information	Case Description, opening paragraph (background of complex family dynamics and socioeconomic adversity from an early age, with a recent financial stressor; further detail withheld to protect confidentiality).
	5d	Relevant past interventions with outcomes	Not applicable: no prior treatment for this condition; first presentation of this kind
**Clinical Findings**	6	Describe significant physical examination (PE) and important clinical findings	Case Description, second paragraph (vital signs, GCS 13/15, systemic examination)
**Timeline**	7	Historical and current information from this episode of care organized as a timeline	Table 2, Clinical Timeline of Episode of Care
**Diagnostic Assessment**	8a	Diagnostic testing (such as PE, laboratory testing, imaging, surveys)	Diagnostic Assessment section, first paragraph; Table 1, Complete Laboratory and Diagnostic Panel
	8b	Diagnostic challenges (such as access to testing, financial, or cultural)	Discussion, Strengths and Limitations (quantitative serum melatonin assays not routinely available)
	8c	Diagnosis (including other diagnoses considered)	Diagnostic Assessment section, first paragraph (negative comprehensive co-ingestant screen used to confirm isolated melatonin exposure)
	8d	Prognosis (such as staging in oncology) where applicable	Diagnostic Assessment section (Poison Severity Score Grade 1, Minor); Table 1
**Therapeutic Intervention**	9a	Types of therapeutic intervention (such as pharmacologic, surgical, preventive, self-care)	Diagnostic Assessment/Therapeutic Intervention section, second paragraph (IV fluids, dextrose, pantoprazole, ondansetron, supportive monitoring)
	9b	Administration of therapeutic intervention (such as dosage, strength, duration)	Diagnostic Assessment/Therapeutic Intervention section<br>Same paragraph (specific doses and administration schedule provided)
	9c	Changes in therapeutic intervention (with rationale)	Not applicable: no change in therapeutic approach was required during the admission
**Follow-up and Outcomes**	10a	Clinician and patient-assessed outcomes (if available)	Follow-up paragraph (somnolence fully resolved, GCS 15, medically cleared)

Topic	Item	Checklist Item Description	Reported in Manuscript (Section)
	10b	Important follow-up diagnostic and other test results	Follow-up paragraph (repeat VBG confirming resolution of acidosis; normalization of sodium and WBC)
	10c	Intervention adherence and tolerability (How was this assessed?)	Not applicable in the pharmacovigilance sense — supportive inpatient care was administered and tolerated without complication
	10d	Adverse and unanticipated events	None reported; supplemental oxygen threshold was not met and no adverse events occurred
**Discussion**	11a	A scientific discussion of the strengths AND limitations associated with this case report	Discussion, “Strengths and Limitations” subsection
	11b	Discussion of the relevant medical literature with references	Discussion, “Comparison with the Published Case Literature,” “Population-Level Suicidality Signals,” “Toxicokinetics and Product-Content Variability,” and “Pediatric Safety Signals and Forensic Considerations” subsections; Table 3; Figure 1
	11c	The scientific rationale for any conclusions (including assessment of possible causes)	Discussion, throughout, and “Clinical Take-Away” subsection
	11d	The primary “take-away” lessons of this case report (without references) in a one paragraph conclusion	Discussion, “Clinical Take-Away” subsection, closing paragraph
**Patient Perspective**	12	The patient should share their perspective in one to two paragraphs on the treatment(s) they received	See 'Patient Perspective' section of the manuscript. Given the patient's minor status, recent suicide attempt, and ongoing treatment at the time of writing, a first-person reflective statement was not sought, as this was considered inappropriate to the clinical context.
**Informed Consent**	13	Did the patient give informed consent? Please provide if requested	Yes. Written informed consent for publication was obtained from the patient's parent (see “Patient Consent for Publication” section of the manuscript).
